# BETWEEN ISLANDS—A CREATIVE PRACTICE AND INTERGENERATIONAL CONTACT INTERVENTION

**DOI:** 10.1093/geroni/igad104.0086

**Published:** 2023-12-21

**Authors:** Ashley Lytle, Nancy Nowacek

**Affiliations:** Stevens Institute of Technology, Hoboken, New Jersey, United States; Stevens Institute of Technology, Hoboken, New Jersey, United States

## Abstract

Addressing ageism is a serious social issue in need of targeted, empirically based solutions (WHO, 2021). Facilitating intergenerational contact is one promising means of reducing ageism (Lytle et al., 2020). Between Islands were a creative practice and intergenerational contact intervention designed to promote creativity and intergenerational connection between younger and older adults while challenging stereotypes about aging. Over a four-week intervention, younger and older adults meet virtually (on zoom) for an hour each Wednesday to learn, create, and discuss with one another. Before the intervention began, participants received two 2.5 lb blocks of air-drying clay, paints, and paintbrushes. During the four-week intervention, participants engaged in creative practice (designed, sculpted, and decorated two hand-weights out of the provided clay). In addition, participants learned about the history of hand-weights and circuit training and got to know one another through group-based discussion as well as smaller group prompts and breakout rooms. Prior to the start of the intervention, baseline attitudes and stereotyping toward older adults were assessed among the younger adult participants. Attitudes and stereotypes were again measured after the intervention concluded. Younger adults reported a significant decrease in benevolent ageism and intergenerational tension after the Between Islands intervention. In addition, themes that emerged from the open-ended responses illustrated that the intervention challenged younger adults’ stereotypes about older adults, encouraged recognition of similarities between younger and older adults, and facilitated the connection between participants.

